# Visual symptoms in postural tachycardia syndrome: An investigation of position‐dependent visual exploration

**DOI:** 10.1111/ene.16507

**Published:** 2024-10-27

**Authors:** Belén Rodriguez, Lynn Pantano, Tobias Nef, René M. Müri, Werner J. Z'Graggen

**Affiliations:** ^1^ Department of Neurosurgery, Inselspital Bern University Hospital, University of Bern Bern Switzerland; ^2^ Department of Neurology, Inselspital Bern University Hospital, University of Bern Bern Switzerland; ^3^ Gerontechnology and Rehabilitation Group, ARTORG Center for Biomedical Engineering Research University of Bern Bern Switzerland

**Keywords:** autonomic dysfunction, blurred vision, dysautonomia, gaze behaviour, visual dysfunction

## Abstract

**Background and Purpose:**

Patients with postural tachycardia syndrome report position‐dependent visual symptoms. Despite their impact on daily life, these symptoms have remained largely unexplored in research. The aim of this study was to investigate the nature of visual symptoms in postural tachycardia syndrome and possible underlying pathophysiological mechanisms.

**Methods:**

Fifteen patients with postural tachycardia syndrome and 15 healthy controls were included in the study. Through a comprehensive array of measurements, including haemodynamics, subjective symptom assessments, eye movement tracking and pupil diameter analysis, participants were assessed during free image exploration in both supine and 60° head‐up tilt positions.

**Results:**

During head‐up tilt, patients showed a decreased number and duration of fixations, as well as a decreased number, peak velocity and amplitude of saccades compared to the supine position and the control group. This reduction in visual exploration occurred primarily in the peripheral field of view and coincided with the occurrence of subjective visual symptoms. No significant differences in the saccade main sequence were observed between the two groups in either body position.

**Conclusions:**

Patients with postural tachycardia syndrome have a reduced exploration of the peripheral field of view when in an upright body position, potentially leading to tunnel vision. Since the normality of the saccade main sequence in patients combined with the focus on the centre of the field of view and the lower saccade amplitudes points to an intact brainstem function, the decrease in peripheral visual exploration may be attributed to a position‐dependent dysfunction of the frontal eye field.

## Graphical abstract

The results of the present study demonstrate as a primary finding a position‐dependent alteration of visual exploration behaviour in patients with postural tachycardia syndrome. Specifically, a reduced peripheral visual exploration in the upright position became apparent, possibly leading to tunnel vision in patients. This phenomenon represents a potential explanation for the occurrence of some of the visual symptoms in postural tachycardia syndrome and is probably associated with a position‐dependent functional disturbance of the frontal lobe, especially the frontal eye field. The figure shows an exemplary visualization of gaze behaviour of patients with postural tachycardia syndrome and control subjects in the supine and upright body positions. The warmth of the colour and size of the spots represent the number and duration of fixation; that is, the warmer the colour, the more and longer participants fixated the spot.

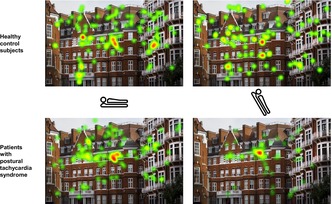

## INTRODUCTION

Postural tachycardia syndrome (POTS) is a chronic disorder of the autonomic nervous system that affects the function of multiple organ systems [1]. This most common subtype, the ‘neuropathic’ POTS, is thought to be caused by peripheral sympathetic denervation or dysfunction leading to increased venous pooling and relative central hypovolaemia [2, 3]. Visual symptoms are common in POTS; 69% of patients report experiencing them on a daily basis and that they lead to the greatest restrictions in everyday life, along with fatigue and orthostatic intolerance [4, 5]. Patients often describe their visual symptoms as blurry or tunnel vision [6]. Despite their high prevalence, visual symptoms have received limited research attention. Hence, the pathophysiology of visual symptoms in POTS is largely unclear.

Visual symptoms can have various causes; two of the most important pathophysiological mechanisms are disturbed eye movements and/or pupillary function [7, 8]. Eye movements are divided into gaze‐shifting (saccades) and gaze‐holding (fixation) mechanisms [9]. Anatomically, the frontal eye field and the paramedian pontine reticular formation play a key role for ocular movements. The latter is located in the brainstem and enables the initiation of horizontal saccades [9]. Situated in the frontal lobe and forming a component of the premotor cortex, the frontal eye field is involved in preparing and initiating intentional eye movements and has an impact on higher cognitive functions, such as coupling visuo‐spatial attention and eye movement, often referred to as overt attentional orienting. The autonomic nervous system influences numerous ocular functions such as the control of the pupil size, accommodation of the lens, regulation of blood flow in the eye and intraocular pressure [10]. The close control of functions of the eye by the autonomic nervous system makes it likely that these may be impaired in patients with POTS.

The aim of this study was to investigate visual exploration and pupillary function of patients with POTS in order to better understand the characteristics of visual symptoms. For this purpose, eye movements during free visual exploration and pupillary function were studied in patients with POTS and healthy subjects in the supine position and during head‐up tilt (HUT). It is hypothesized that patients with POTS show position‐dependent differences in eye movement patterns and pupillary diameter compared to healthy subjects.

## METHODS

### Participants

All study procedures were performed between April and October 2023 at the University Hospital Bern, Switzerland. This study received ethical approval (Kantonale Ethikkommission Bern, Switzerland; project‐ID 2022–00592), and was carried out and reported in accordance with the Declaration of Helsinki and its amendments. All participants gave written informed consent. Fifteen patients with neuropathic POTS and 15 healthy control subjects were included. Exclusion criteria for all participants were pregnancy (ruled out by a pregnancy test performed prior to start of the experiment), breast feeding and age ≤18 or ≥60 years. Additional exclusion criteria for control subjects included untreated hypertension, vasovagal syncope in medical history and intake of vasoactive medication. All participants had normal or corrected‐to‐normal vision. Healthy volunteers were recruited at the University of Bern using flyers. Diagnosis of neuropathic POTS was made on the basis of a series of examinations described in previous publications [11, 12, 13]. Participants were not allowed to eat or drink for at least 6 h before the examination. All vasoactive and psychoactive medication was stopped at five half‐lives prior to testing.

## MATERIAL

### Cardiovascular autonomic function testing

Participants were positioned comfortably in the supine position on a tilt table. Throughout the experimental procedure, the Finapres® Portapres device (Finapres Medical Systems BV, Arnhem, the Netherlands) was used to continuously record heart rate and beat‐to‐beat blood pressure from the right hand. Additionally, a three‐lead electrocardiogram and breathing rates were recorded. Intermittent brachial blood pressure was measured on the left arm using a Mindray VS‐900 sphygmomanometer (ITRIS Medical AG, Spreitenbach, Switzerland). HUT testing was conducted with a tilt angle of 60°.

### Symptom assessment

The following 10 subjective symptoms of the participants were assessed twice during the examination: headache, dizziness, nausea, feeling of weakness, palpitations, light‐headedness, shortness of breath, fatigue, concentration difficulties, blurred vision. Participants rated the symptoms on a visual analogue scale from zero to 10, with zero being the absence of the symptom and 10 the worst imaginable severity of the symptom (maximum sum score 100).

### Visual exploration

Eye movements were measured during free exploration of images using an eye tracking device with a sampling rate of 120 Hz (Tobii Pro X3‐120 eye tracker, Tobii Technology, Sweden) attached to a 24‐inch monitor, which was angled at 15° and fixed to the tilt table to ensure a continuous viewing distance of 60 cm. Tobii Pro Lab was used for image presentation as well as for eye movement data acquisition and raw data analysis. A total of three visual exploration tasks were performed. During each task, participants were shown a series of 20 different photographs of nature and architecture, which had been previously validated and used in similar studies [14, 15]. The presentation order of the image sets was counterbalanced amongst participants, and the order of images within each set was randomized by the software. Before each task, a 9‐point calibration was conducted, followed by a validation procedure. If the validation procedure indicated data loss greater than 10%, validation was repeated. Each task started with a central fixation marker displayed for 1.5 s, followed by an image presented for 7 s. In between images, the central fixation marker reappeared for 1.5 s. The duration of a visual exploration task was 4 min including calibration and validation. Participants were instructed to freely explore the images, as if they were looking at photographs in a photo album, and to fixate the central fixation marker when it was displayed. The following parameters were assessed: number of fixations, cumulative fixation duration (ms), number of saccades, maximum saccade peak velocity (deg/s) and cumulative saccade amplitude (deg).

### Pupillary assessment

During each exploration task the pupil diameters (mm) of both eyes were continuously measured by the eye tracker. The average pupil diameter of both eyes was used for the analysis. The pupillary light reflex was repeatedly measured in both eyes with an NPi‐200 portable infrared pupilometer (NeurOptics, Irvine, CA, USA). Pupillary assessments were always carried out on the right eye first. The parameter assessed by the pupilometer was pupillary constriction velocity (mm/s).

## STUDY PROTOCOL

**FIGURE 1:**
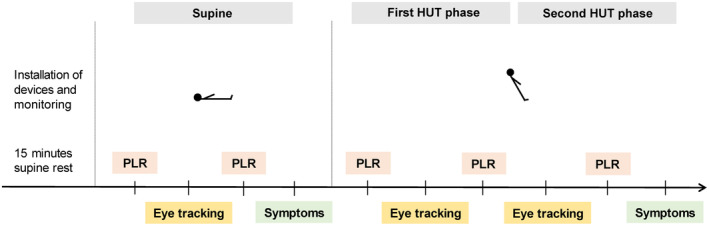
Study protocol. Participants completed three visual exploration tasks (eye tracking) in total, once in the supine position and twice during 60° HUT. The PLR was taken five times, each before and after a visual exploration task. Once in the supine position and once at the end of the HUT phase, all participants rated their symptoms of orthostatic intolerance. HUT, head‐up tilt; PLR, pupillary light reflex.

Figure 1 provides a detailed illustration of the study protocol. All study procedures were conducted in a light‐ and temperature‐controlled environment (Autonomic Laboratory of the University Hospital Bern). Participants remained in the HUT position for 10 min; the total duration of the entire study procedure was 60 min.

## STATISTICAL ANALYSIS

Statistical analyses were performed using R, version 4.3.1, through R studio interface and SPSS Statistics 25.0 (IBM, Armonk, NY, USA). Parametric tests were performed for normally distributed data and non‐parametric tests for non‐normally distributed variables. Group differences in patient characteristics were assessed using a two‐tailed Student's *t* test for independent samples. Group and position‐dependent differences in haemodynamic data were assessed using 2 × 2 repeated‐measures analyses of variance (ANOVA) with *post hoc* Bonferroni correction for multiple comparisons. The factors in the ANOVA were (i) group (patients; healthy control subjects) and (ii) position (supine; HUT). Group and position‐dependent differences in symptom ratings were analysed using the Mann–Whitney *U* test and Wilcoxon signed‐rank test, respectively.

To analyse the eye tracking parameters, 2 × 3 repeated‐measures ANOVA with *post hoc* Bonferroni correction for multiple comparisons was conducted. The parameters analysed were mean values of the individual parameters for each image over the 20 images of a set. The factors were (i) group (patients; healthy control subjects) and (ii) position (supine; first HUT phase; second HUT phase). For the analysis of the constriction velocity in the pupillary light reflex, a 2 × 5 repeated‐measures ANOVA with *post hoc* Bonferroni correction was conducted. The factors were (i) group (patients; controls) and (ii) position (supine baseline; just before HUT; immediately after HUT; after 5 min of HUT; at the end of HUT). The saccade main sequences as a representation of the relationship between peak velocity and amplitude of (singular) saccades were modelled applying a square root equation for each position (supine; first HUT phase; second HUT phase). Mixed effect models were applied to consider the multiple measurements for each participant. The saccade amplitude was multiplied by a constant, set to 98 as done in Lebedev et al. (1996) [16].

To assess the distribution of fixations, three equally sized, horizontally aligned areas of interest covering the entire height of the screen (left image third, centre and right image third) were defined. For each participant, the number and cumulative duration of fixations was calculated for the three areas separately; the values of the left and right areas were cumulated to the variable ‘periphery’, thus covering the two outer thirds of the image, and compared to the central area. The parameters were analysed with a 2 × 2 × 3 repeated‐measures ANOVA with *post hoc* Bonferroni correction. The factors were (i) group (patients; healthy control subjects); (ii) area of interest (centre; periphery) and (iii) position (supine; first HUT phase; second HUT phase). The centre of gravity of all fixations on the horizontal axis (*x*‐axis) was analysed by calculating the mean *x*‐coordinates of all fixations and the standard deviation for each position separately (supine; first HUT phase; second HUT phase). 2 × 3 repeated‐measures ANOVA with *post hoc* Bonferroni correction were conducted to analyse possible changes of the centre of gravity and the standard deviation. The factors were the same as in the first ANOVA. Pearson's correlations were performed to assess potential associations between the pupil diameter and haemodynamic data as well as between the distribution (standard deviation) of the centre of gravity of fixations and haemodynamic data and symptom ratings. Group data are reported as mean (± standard deviation) unless otherwise stated. A two‐tailed *p* value ≤0.05 was defined as statistically significant.

Since this was the first study examining eye movements and pupillary function in this patient population, the sample size had to be estimated (using G*Power 3.1.9.2) for a repeated‐measures ANOVA. Based on the results of similar previous studies implementing a free exploration task [17, 18, 19], a medium effect size was specified, that is, *f* = 0.3, *α* = 0.05, 1 − *β* = 0.90, correlation amongst measures 0.5, sphericity correction 1, number of groups 2 and number of measurements 3. This resulted in a final total required sample size of *n* = 26.

## RESULTS

### Participants characteristics, haemodynamic data and symptom rating

Table 1 summarizes the participants’ demographic data, haemodynamic data and symptom ratings. The mean heart rate increase upon HUT was more pronounced in patients with POTS than in healthy subjects (+30.93 ± 9.94 vs. +19.2 ± 8.66 bpm; *p* = 0.002). Patients had significantly higher breathing rates during HUT compared to the supine position (*p* = 0.035), and reported a significant increase of the cumulative symptom score (*p* < 0.001) and of ‘blurred vision’ (*p* = 0.024) upon HUT.

**TABLE 1:** Patient characteristics, haemodynamic data and symptom ratings.

		HC (*n* = 15)	POTS (*n* = 15)	*p* value*
Age		25.20 ± 3.93	27.60 ± 5.84	0.197
Sex, female (*n*)		15	15	‐
Supine	Systolic blood pressure (mmHg)	103.13 ± 5.01	113.53 ± 9.49	<0.001
	Diastolic blood pressure (mmHg)	65.20 ± 5.34	74.40 ± 5.80	<0.001
	Heart rate (bpm)	59.20 ± 7.84	68.87 ± 12.00	0.014
	Breathing rate (brpm)	16.10 ± 2.08	15.20 ± 2.29	0.303
	Symptom rating	3.27 ± 2.55	9.20 ± 10.28	0.056
	Blurred vision	0.13 ± 0.52	0.67 ± 1.29	0.345
HUT	Systolic blood pressure (mmHg)	107.93 ± 10.15	124.00 ± 14.71	0.002
	Diastolic blood pressure (mmHg)	74.47 ± 6.92	83.40 ± 7.46	0.002
	Heart rate (bpm)	78.40 ± 8.04	99.80 ± 17.06	<0.001
	Breathing rate (brpm)	16.20 ± 2.56	17.60 ± 4.8	0.344
	Symptom rating	4.33 ± 4.05	29.67 ± 20.05	<0.001
	Blurred vision	0.07 ± 0.26	1.87 ± 2.13	0.008

*Note*: Data are reported as mean ± SD if not differently indicated. Heart rate, blood pressure, breathing rates and symptom ratings were assessed in the supine position just before HUT and 10 min after the start of HUT.

Abbreviations: bpm, beats per minute; brpm, breaths per minute; HC, healthy controls; HUT, head‐up tilt; POTS, postural tachycardia syndrome.

* *p* values refer to group differences.

### Visual exploration

**FIGURE 2:**
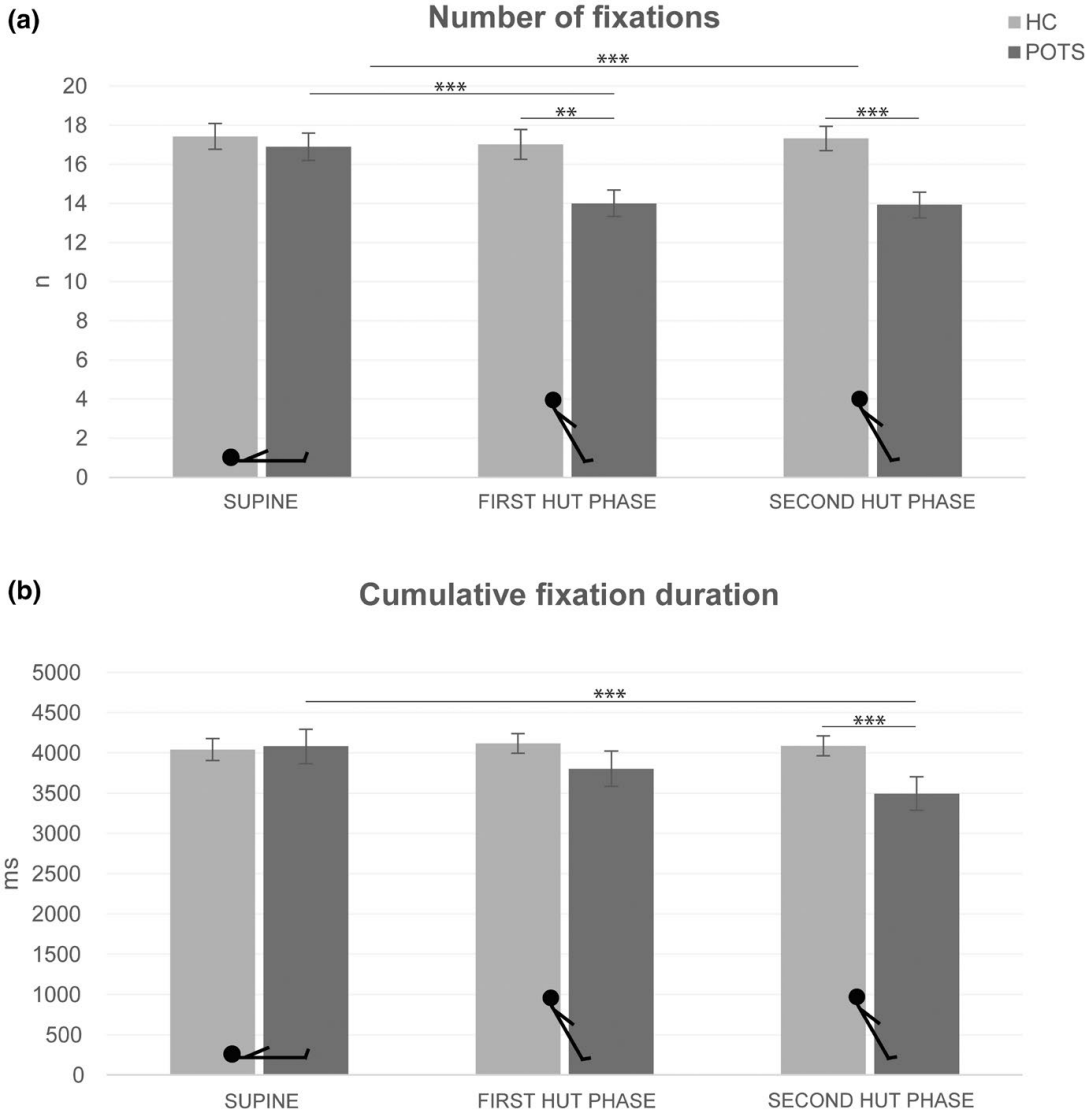
Results of eye tracking during visual exploration: fixation metrics. (a) Bar graphs showing the number of fixations of patients with POTS (dark grey bars) and control subjects (light grey bars) in the supine and 60° HUT positions (first and second HUT phase). (b) The same is shown for cumulative fixation duration. For number of fixations, there were main effects of position (*F*(1, 28) = 6.08, *p* = 0.007) and group (*F*(1, 28) = 8.32, *p* = 0.007) as well as a significant interaction of position × group (*F*(1, 28) = 4.29, *p* = 0.024). For cumulative fixation duration, the analysis showed a main effect of position (*F*(1, 28) = 3.83, *p* = 0.034) and an interaction of position × group (*F*(1, 28) = 5.20, *p* = 0.012). Values are given as mean ± SEM. HC, healthy controls; HUT, head‐up tilt; POTS, postural tachycardia syndrome. **p ≤ 0.01, ***p ≤ 0.001.

**FIGURE 3:**
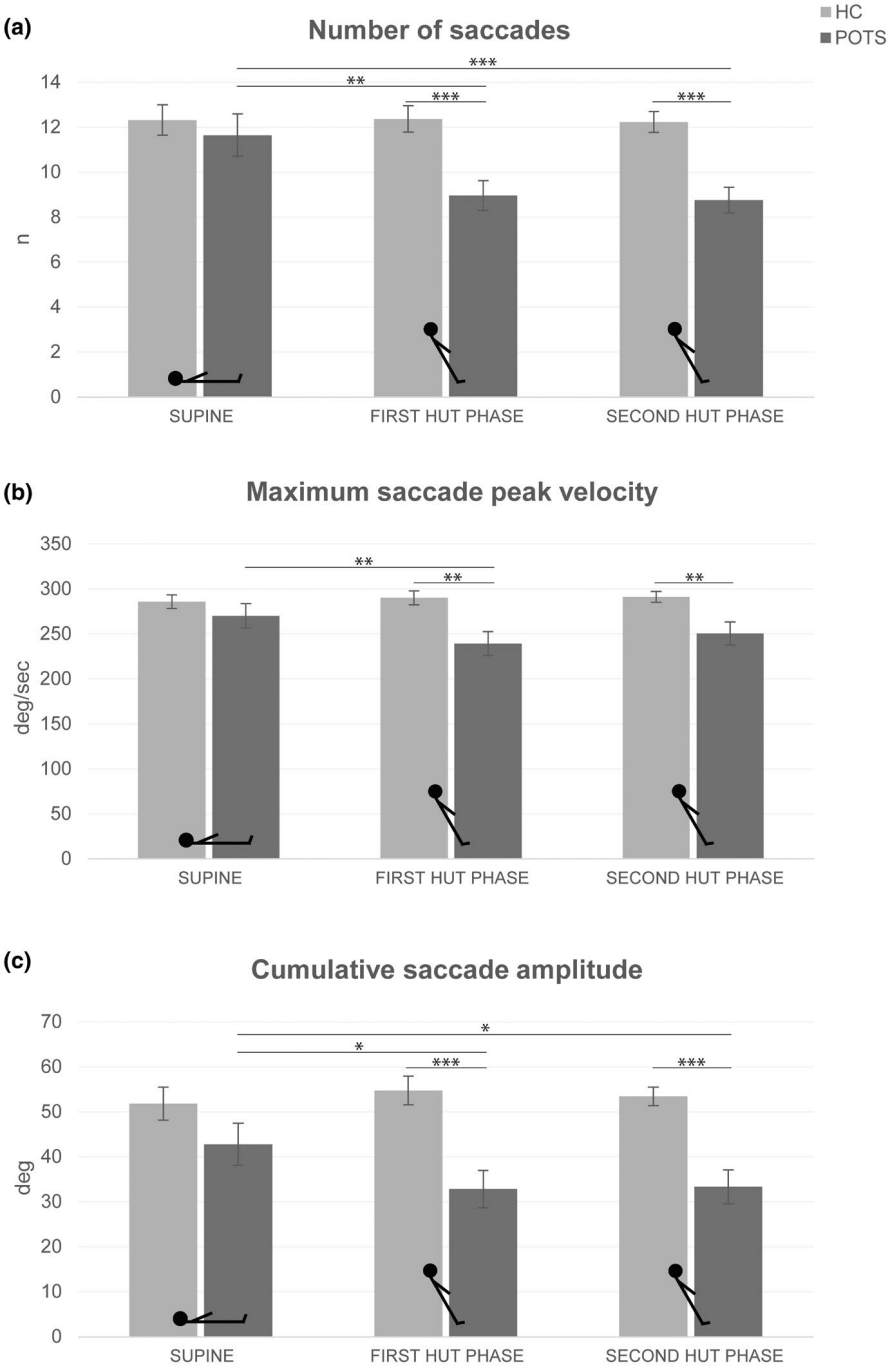
Results of eye tracking during visual exploration: saccade metrics. (a) Bar graphs showing the number of saccades of patients with POTS (dark grey bars) and control subjects (light grey bars) in the supine and 60° HUT positions (first and second HUT phase). (b) The same is shown for maximum saccade peak velocity and (c) cumulative saccade amplitude. The analysis of number of saccades showed significant main effects for position (*F*(1, 28) = 4.87, *p* = 0.016) and group (*F*(1, 28) = 10.71, *p* = 0.003) as well as an interaction of position × group (*F*(1, 28) = 4.48, *p* = 0.021). For maximum peak velocity of saccades, there was a main effect for group (*F*(1, 28) = 7.34, *p* = 0.011) and a significant interaction of position × group (*F*(1, 28) = 3.84, *p* = 0.034). For cumulative amplitude of saccades, the analysis showed a significant main effect for group (*F*(1, 28) = 14.43, *p* < 0.001) and an interaction of position × group (*F*(1, 28) = 3.69, *p* = 0.038). Values are given as mean ± SEM. HC, healthy controls; HUT, head‐up tilt; POTS, postural tachycardia syndrome. *p ≤ 0.05, **p ≤ 0.01, ***p ≤ 0.001.

**FIGURE 4:**
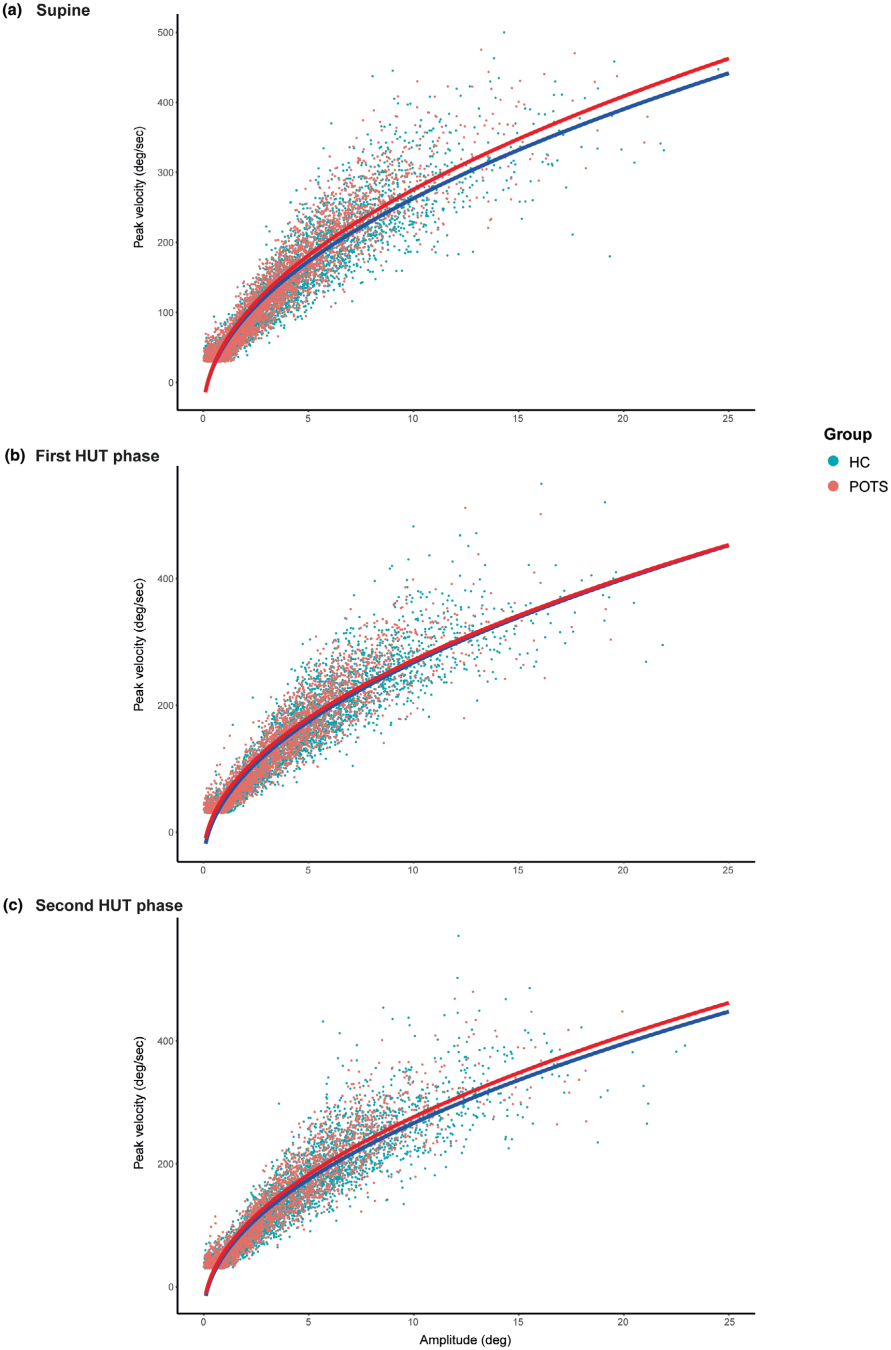
Saccade main sequence. (a) Graphs showing the relationship between peak velocity and amplitude of saccades (saccade main sequence) of patients with POTS (red) and control subjects (blue) in the supine position. (b) The same is shown for the 60° HUT position (first HUT phase) and in (c) for the 60° HUT position (second HUT phase). HC, healthy controls; HUT, head‐up tilt; POTS, postural tachycardia syndrome.

Numerical values of the analyses of the visual exploration are shown in Table S1, and the results are reported and illustrated in Figures 2 and 3. Overall, patients showed a reduced visual exploration during HUT compared to their exploration in the supine position as well as compared to the control group, which showed no position‐dependent changes of visual exploration. This manifested in a reduction of the number and cumulative duration of fixations as well as the number, maximum peak velocity and cumulative amplitude of saccades. The reduction in saccade amplitudes during HUT in patients was reflected in the saccade main sequences (Figure 4). During both HUT phases, there were fewer data points with larger amplitudes in patients than in the supine position and compared to healthy subjects. The square root equation models showed that there were no relevant differences in the slope between patients with POTS and healthy subjects in both body positions. The estimated parameter (POTS vs. healthy subjects) was 6.6 (confidence interval −0.5 to 13.7) in the supine position; 6.2 (confidence interval −0.5 to 12.9) in the first HUT phase; and 6.9 (confidence interval −0.4 to 14.2) in the second HUT phase.

#### Areas of interest and centre of gravity

**FIGURE 5:**
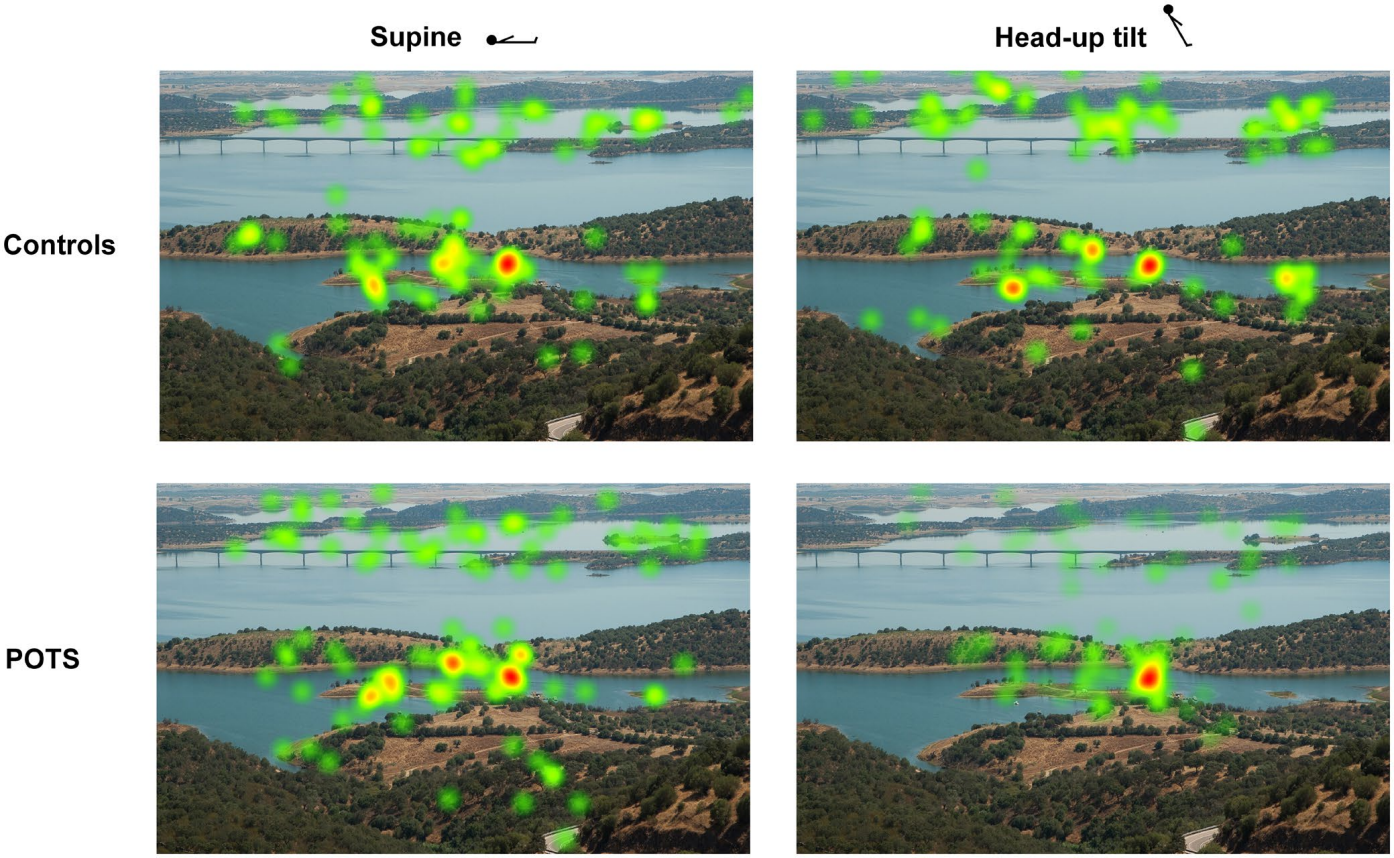
Heat maps: illustration of one image. Exemplary visualization of gaze behaviour of patients with POTS and control subjects in the supine position and during 60° HUT (second HUT phase). Each image contains the cumulative fixations of five participants who were shown the image in the same position. The warmth of the colour and size of the spots represent the number and duration of fixation; that is, the warmer the colour, the more and longer participants fixated the spot. HUT, head‐up tilt; POTS, postural tachycardia syndrome.

**FIGURE 6:**
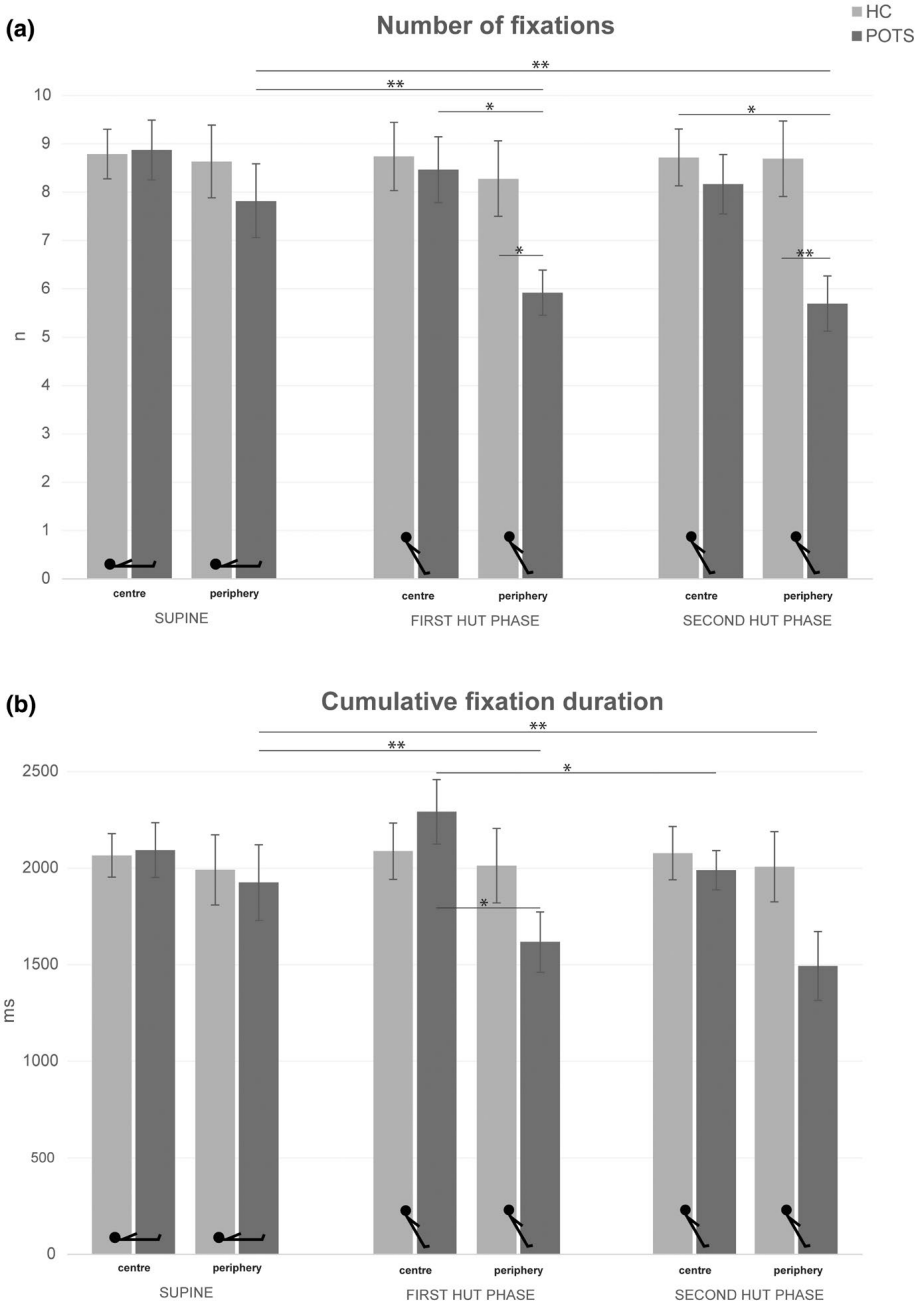
Results of eye tracking during visual exploration: areas of interest. (a) Bar graphs showing the number of fixations to the centre and periphery of the field of view, respectively, of patients with POTS (dark grey bars) and control subjects (light grey bars) in the supine and 60° HUT positions (first and second HUT phase). (b) The same is shown for cumulative fixation duration. The analysis of number of fixations in each area of interest (centre vs. periphery) showed main effects of position (*F*(1, 28) = 5.10, *p* = 0.013) and group (*F*(1, 28) = 7.57, *p* = 0.010) as well as a significant interaction of position × group (*F*(1, 28) = 3.88, *p* = 0.033). For cumulative fixation duration, there was a significant main effect for position (*F*(1, 28) = 4.11, *p* = 0.028) and an interaction of position × group (*F*(1, 28) = 4.41, *p* = 0.022). Values are given as mean ± SEM. HC, healthy controls; HUT, head‐up tilt; POTS, postural tachycardia syndrome. *p ≤ 0.05, **p ≤ 0.01.

Numerical values of the analyses of the areas of interest are shown in Table S2, and the results are reported and illustrated in Figures 5 and 6. The analyses of the areas of interest specified that the position‐dependent reduction of visual exploration in patients with POTS was due to a decrease of the visual exploration to the periphery of the field of view. The analysis of the centre of gravity (mean *x*‐coordinate of fixations) showed no main effects or interactions and no pairwise comparisons were significant (healthy subjects, supine 0.51, first HUT phase 0.51, second HUT phase 0.51; patients with POTS, supine 0.51, first HUT phase 0.51, second HUT phase 0.50). For the scattering of fixations around the centre of gravity (standard deviation of mean *x*‐coordinate of fixations), there were again no significant main effects or interaction, but pairwise comparisons showed that in patients with POTS the standard deviation decreased from the supine position to the first (*p* = 0.021) and second (*p* = 0.031) HUT phase (supine 0.20; first HUT phase 0.18; second HUT phase 0.18). There were no such changes in healthy subjects (supine 0.20; first HUT phase 0.20; second HUT phase 0.20). The correlation analyses revealed no associations between the centre of gravity parameters and any of the clinical or haemodynamic variables.

#### Pupillary assessment

The analysis of the mean pupil diameter during eye tracking showed a main effect of position (*F*(1, 28) = 14.86, *p* < 0.001) due to an increase in pupil diameter in patients during HUT compared to the supine position (supine 3.75 [± 0.85] mm vs. first HUT phase 3.98 [± 0.92] mm, *p* < 0.001; and vs. second HUT phase 3.93 [± 0.88] mm, *p* < 0.001). In healthy subjects, there were no positional changes in pupil diameter (supine 3.39 [± 0.32] mm; first HUT phase 3.49 [± 0.28] mm; second HUT phase 3.45 [± 0.32] mm). The mean pupil diameter correlated with systolic blood pressure in the supine position (*r* = 0.421; *p* = 0.020), and with heart rate in the supine position (*r* = 0.371; *p* = 0.043) and during the second HUT phase (*r* = 0.604; *p* < 0.001). The analysis of the pupillary light reflex (constriction velocity) revealed no significant main effects or interactions (healthy subjects supine baseline 3.66 [± 0.52] mm/s, just before HUT 3.71 [± 0.39] mm/s, immediately after HUT 3.5 [± 0.48] mm/s, after 5 min of HUT 3.57 [± 0.35] mm/s, at the end of HUT 3.5 [± 0.37] mm/s; POTS supine baseline 3.5 [± 0.51] mm/s, just before HUT 3.25 [± 0.52] mm/s, immediately after HUT 3.28 [± 0.44] mm/s, after 5 min of HUT 3.38 [± 0.66] mm/s, at the end of HUT 3.38 [± 0.6] mm/s).

## DISCUSSION

This study investigated visual exploration and pupillary function of individuals with POTS. In summary, the results indicate a position‐dependent decrease in visual exploration of the peripheral areas of the field of view.

In the supine position, patients with POTS had a similar visual exploration to healthy controls, whereas, during standing, patients with POTS showed fewer fixations and a lower cumulative fixation duration as well as fewer, slower and smaller saccades compared to the supine position and to the control group. Healthy controls did not show any position‐dependent changes in explorative behaviour. Whilst the centre of gravity of all fixations did not change in patients with POTS during standing compared to the supine position and to healthy controls, the distribution of fixations narrowed significantly, resulting in fewer fixations in the peripheral areas of the field of view. These results suggest the occurrence of a tunnel vision phenomenon in POTS when they are upright, a symptom that is often described by patients [6]. Peripheral vision plays a crucial role in creating and maintaining an accurate mental map of the surrounding environment for effective navigation, and serves to explore and direct attention in a scene in order to prioritize the analysis of regions relevant to a task or an activity [20, 21]. A reduced peripheral vision has an impact on many aspects of daily life in patients with POTS and corresponds to commonly reported complaints, for example difficulties in performing tasks where it is necessary to maintain a visual overview of a situation or navigating in a lively surrounding.

The saccade main sequence in patients with POTS did not differ from healthy subjects in either body position, indicating an intact function of the paramedian pontine reticular formation in the brainstem [9]. In addition, the efferent part of the pupillary light reflex, assessed by the constriction velocity, was normal in POTS. Since this part of the pupillary light reflex is mainly controlled by the upper brainstem, this further points to the integrity of the function of this brain region [22]. It is therefore possible that the changes in gaze behaviour in patients with POTS described above are related to a position‐dependent disturbance of the function of the frontal eye field. This link to the frontal eye field is strengthened by the findings of Cazzoli et al. [23], who produced similar findings—relative neglect of the peripheral field of vision and the lower saccade amplitudes—by inhibitory transcranial brain stimulation over the right frontal eye field in healthy individuals. Specifically, they found a distinct narrowing of overt visual attention deployment, resulting in an extended cumulative fixation duration in the central region of the stimuli and a reduced cumulative fixation duration in both the right and left peripheral fields of vision. It is therefore postulated that the reduction in peripheral visual exploration in patients with POTS is possibly due to an altered function of the frontal eye field, which is dependent on the body position.

Why such a dynamic dysfunction of the frontal eye field may occur in POTS remains unclear. It has been shown that patients with neuropathic POTS experience an increased activation of the sympathetic nervous system, possibly as a compensatory mechanism to counterbalance the insufficient peripheral vasoconstriction [24]. In the present study, the increased blood pressure and larger pupil in patients with POTS compared to the control group are probably due to this elevated sympathetic activity. Empirical evidence suggests an augmented release of norepinephrine, particularly evident during orthostasis [24, 25, 26]. This increased activation of the sympathetic nervous system probably leads to a hyperadrenergic ‘stressed’ state in patients with POTS, both peripherally and within the brain [27, 28]. The association between stress in terms of a hyperadrenergic state and the occurrence of altered visual exploration in connection with a possible dynamic disturbance of the frontal eye field is supported by several eye tracking studies that were conducted with healthy individuals under an artificially generated stress situation and that found similar phenomena to the results of the present study. Specifically, Guy et al. [29] found that stressed healthy individuals showed fewer saccadic eye movements and scanned a smaller part of the image. Herten et al. [30] reported that stressed participants showed enhanced memory for central objects, accompanied by longer fixation times and larger fixation amounts on these central objects. It may therefore be speculated that the reduced exploration of peripheral visual field in patients with POTS is related to a dysfunction of the frontal eye field, which may be triggered by a ‘stress‐like state’ due to the increased sympathetic activity when upright. Norcliffe‐Kaufman et al. recently reported that patients with POTS have increased somatic vigilance and may experience an anxiety‐related hyperadrenergic state in an upright position [31], which can trigger hyperventilation and potentially affect cerebral perfusion. The increased sympathetic activity in POTS may therefore be exacerbated by a state of heightened anxiety, which could further increase symptoms.

Certain limitations should be taken into account when considering the findings of the present study. In the assessment of visual symptoms solely ‘blurred vision’ was queried, neglecting tunnel vision and other visual symptoms. Hence, a correlation between the measured visual exploration and the subjective evaluation of specific visual symptoms could not be established. Conclusions regarding the involvement of stress in the development of visual symptoms in patients with POTS are limited as stress levels were not measured in the present study. Since the mean heart rate increase in patients just met the diagnostic criteria for POTS, and the fasting time for all participants was at least 6 h before the examination, it is possible that the results of the study may be different under other conditions. Furthermore, the present study exclusively included female patients with neuropathic POTS, thereby limiting the generalizability of the findings to other POTS cohorts and subtypes.

In conclusion, the results of the present study demonstrate as a primary finding a position‐dependent alteration of visual exploration behaviour in patients with POTS. Specifically, a reduced peripheral visual exploration in the upright position became apparent, possibly leading to tunnel vision in patients. This phenomenon represents a potential explanation for the occurrence of some of the visual symptoms in POTS and may be associated with a position‐dependent functional disturbance of the frontal lobe, especially the frontal eye field.

## AUTHOR CONTRIBUTIONS

**Belén Rodriguez:** Conceptualization; methodology; project administration; writing – original draft; visualization; formal analysis; data curation; investigation. **Lynn Pantano:** Data curation; investigation; formal analysis; writing – original draft; visualization. **Tobias Nef:** Conceptualization; methodology; resources; writing – review and editing. **René M. Müri:** Conceptualization; methodology; supervision; writing – review and editing. **Werner J. Z'Graggen:** Conceptualization; methodology; investigation; supervision; visualization; resources; writing – review and editing.

## CONFLICT OF INTEREST STATEMENT

The authors declare no conflicts of interest.

## Supporting information


Table S1.

Table S2.

## Data Availability

Anonymized data that support the findings of this study are available upon reasonable request to the corresponding author. Applications will be considered on an individual basis, assessing the feasibility and appropriateness of the proposed study and the ability to ensure the required level of data security in accordance with the original ethics committee. Data transfer will be regulated by a material transfer agreement.
