# Supplementary material for: Visual symptoms in postural tachycardia syndrome: An investigation of position‐dependent visual exploration
Source: Eur J Neurol. 2024 Oct 27;32(1):e16507. doi: 10.1111/ene.16507 (PMC11622273; doi:10.1111/ene.16507)
Supplement: Supplementary file 1 — Table S1. [file ENE-32-e16507-s001.docx]

**Table S1 Visual exploration (overall fixation and saccade metrics)**

|  |  | **Supine** | **First HUT phase** | **Second HUT phase** |
| --- | --- | --- | --- | --- |
| Number of fixations | HC | 17.42 (± 2.48) | 17.02 (± 2.81) | 17.32 (± 2.33) |
|  | POTS | 16.90 (± 2.61) | 14.00 (± 2.54) | 13.93 (± 2.47) |
| Cumulative fixation duration (ms) | HC | 4040.68 (± 502.19) | 4118.29 (± 461.21) | 4086.69 (± 463.62) |
|  | POTS | 4080.25 (± 802.00) | 3802.12 (± 820.89) | 3494.90 (± 773.77) |
| Number of saccades | HC | 12.32 (± 2.54) | 12.37 (± 2.21) | 12.23 (± 1.71) |
|  | POTS | 11.65 (± 3.53) | 8.97 (± 2.46) | 8.76 (± 2.12) |
| Maximum saccade velocity (deg/sec) | HC | 285.87 (± 28.62) | 290.16 (± 29.32) | 291.26 (± 22.70) |
|  | POTS | 270.15 (± 50.05) | 239.36 (± 49.48) | 250.59 (48.18) |
| Cumulative saccade amplitude (deg) | HC | 51.84 (± 13.65) | 54.77 (± 11.87) | 53.46 (± 7.72) |
|  | POTS | 42.79 (± 17.45) | 32.83 (± 15.52) | 33.35 (± 14.02) |

Note. Data are reported as mean (± standard deviation). HC, healthy controls, n = 15; POTS, postural tachycardia syndrome, n = 15; HUT, head-up tilt.
