# Supplementary material for: Visual symptoms in postural tachycardia syndrome: An investigation of position‐dependent visual exploration
Source: Eur J Neurol. 2024 Oct 27;32(1):e16507. doi: 10.1111/ene.16507 (PMC11622273; doi:10.1111/ene.16507)
Supplement: Supplementary file 2 — Table S2. [file ENE-32-e16507-s002.docx]

**Table S2 Visual exploration (areas of interest)**

|  |  |  | **Supine** | **First HUT phase** | **Second HUT phase** |
| --- | --- | --- | --- | --- | --- |
| Number of fixations | Centre | HC | 8.79 (± 1.92) | 8.74 (± 2.64) | 8.72 (± 2.20) |
|  |  | POTS | 8.87 (± 2.31) | 8.47 (± 2.52) | 8.17 (± 2.30) |
|  | Periphery | HC | 8.64 (± 2.81) | 8.28 (± 2.89) | 8.69 (± 2.92) |
|  |  | POTS | 7.82 (± 2.83) | 5.92 (± 1.75) | 5.69 (± 2.14) |
| Cumulative fixation duration (ms) | Centre | HC | 2066.61 (± 421.19) | 2088.51 (± 545.34) | 2077.85 (± 515.90) |
|  |  | POTS | 2093.71 (± 529.47) | 2292.26 (± 627.00) | 1989.48 (± 379.49) |
|  | Periphery | HC | 1991.61 (± 680.97) | 2013.49 (± 721.82) | 2007.86 (± 680.05) |
|  |  | POTS | 1926.71 (± 730.79) | 1618.95 (± 578.96) | 1493.78 (± 665.81) |

Note. Data are reported as mean (± standard deviation). HC, healthy controls, n = 15; POTS, postural tachycardia syndrome, n = 15; HUT, head-up tilt.
